# Bioavailability Improvement Strategies for Icariin and Its Derivates: A Review

**DOI:** 10.3390/ijms23147519

**Published:** 2022-07-07

**Authors:** Róbert Szabó, Csaba Pál Rácz, Francisc Vasile Dulf

**Affiliations:** 1 Department of Environmental and Plant Protection, University of Agricultural Sciences and Veterinary Medicine Cluj-Napoca, Calea Mănăştur 3-5, 400372 Cluj-Napoca, Romania; robert.szabo@student.usamvcluj.ro; 2 Faculty of Chemistry and Chemical Engineering, Babeș-Bolyai University of Cluj-Napoca, Arany János 11, 400028 Cluj-Napoca, Romania; csaba.racz@ubbcluj.ro

**Keywords:** bioavailability, complex formation, icariin, icariside II, icaritin, micelle, solubility improvement

## Abstract

In recent years, there has been considerable interest in icariin (ICA) and its derivates, icariside II (ICS) and icaritin (ICT), due to their wide range of potential applications in preventing cancer, cardiovascular disease, osteoporosis, delaying the effects of Alzheimer’s disease, treating erectile dysfunction, etc. However, their poor water solubility and membrane permeability, resulting in low bioavailability, dampens their potential beneficial effects. In this regard, several strategies have been developed, such as pharmaceutical technologies, structural transformations, and absorption enhancers. All these strategies manage to improve the bioavailability of the above-mentioned flavonoids, thus increasing their concentration in the desired places. This paper focuses on gathering the latest knowledge on strategies to improve bioavailability for enhancing the efficacy of icariin, icariside II, and icaritin. We conclude that there is an opportunity for many further improvements in this field. To the best of our knowledge, no such review articles scoping the bioavailability improvement of icariin and its derivates have been published to date. Therefore, this paper can be a good starting point for all those who want to deepen their understanding of the field.

## 1. Introduction

The most plentiful polyphenols occurring in food are the natural flavonoids and their glycosides. To date, more than 15,000 different flavonoids have been identified and isolated from plants [1]. Recently, due to their various health-promoting properties [2], an abundance of food supplements containing those compounds have gained popularity among the general population [3].

Icariin (ICA), icariside II (ICS), and icaritin (ICT) are the main active components of *Epimedii*, a traditional Chinese medicine that was used to treat and prevent numerous health disorders, such as cardiovascular diseases, osteoporosis, or sexual dysfunction [4]. Recently, several studies investigated their potential as drugs against many common health issues. In some cases, their medicinal effects were greater than those of certain currently used drugs [5,6]. For example, in the case of Alzheimer’s disease prevention, they have approximately the same efficacy as donepezil, but they are less toxic [7].

Among various other factors, the low bioavailability of flavonoids remains a major obstacle to increasing their effectiveness [8]. As with other flavonoids, unfortunately, icariin and its derivates also have a low oral bioavailability (~12%) and poor absorption; thus, their clinical applications are limited [9]. Their poor oral bioavailability is caused by low water solubility and membrane permeability, a slow dissolution rate in biological fluids, etc. Compared to other routes, oral administration assures greater comfort, less pain, and, most importantly, higher patient compliance [10]. Therefore, more attention should be paid to overcoming this hurdle.

Motivated by all those factors above, this paper proposes a review of improvements to the bioavailability of icariin and its derivates. The first part focuses on the importance of icariin and its derivates, due to their wide-ranging therapeutic effects. Furthermore, the significance of the bioavailability of poorly water-soluble drugs is discussed. The main part of the paper deals with the current bioavailability improvement strategies regarding icariin, icariside II and icaritin.

## 2. Icariin and Its Derivates

In ancient China and thence, elsewhere, the herbal medicine *Herba Epimedii* has been used as a tonic due to its powerful anti-rheumatic properties and aphrodisiac effects [11]. According to the Chinese pharmacopeia, the main active component of the *Epimedium* sp. was found to be icariin and icariside II, the last being also called baohouside-I [12]. With mass spectrometry methods, the epimedins A–C and sagittatoside B have been established as other active components of the *Herba Epimedii* [13]. Several metabolism studies performed by means of HPLC methods have found that the intestinal flora metabolized the icariin, converting it mainly into icariside II, icaritin, and desmethylicaritin [14]. It was demonstrated that ICS is the secondary metabolite because its content exceeded every other active agent [15].

### 2.1. Extraction and Preparation

A vast amount of ICA and ICS can be successfully extracted from *Epimedium koreanum*, another herb often used in traditional Chinese medicine. As a result of purification with a two-phase chloroform-methanol-water solvent system from 200 mg of extract, 46.5 mg of ICA, and 17.7 mg of ICS were obtained. By means of high-speed counter-current chromatography, a single-step separation process resulted in a high-purity (<98%) product [16]. The low content of ICS found in the extraction showed the necessity for another method capable of extracting it with a significantly higher yield. One of the most viable solutions for preparing ICS is the enzymatic hydrolysis of the ICA. With this process, 98% of the icariin was transformed into ICS. This technique consists of a reaction between ICA and β-glucosidase and an extraction with ethyl acetate [17]. In order to prepare a higher amount of ICS, a laboratory pilot scale (500 mL) of enzymatic hydrolysis was successfully developed, which resulted in a 91% yield [18]. Recent studies found an eco-efficient industrial preparation to produce ICS from ICA via enzymatic hydrolysis. The main difference in preparation was achieved by substituting the β-glucosidase enzyme with β-glucanase. In this process, the conversion ratio of ICA was 99%, which exceeded the previous ratios [19]. Viable industrial production of icaritin originated from the discovery of a catalytic enzyme, a glycosidase from *Aspergillus nidulans*, which made possible the biotransformation of epimedin C into ICT [20]. Further studies revealed possibilities for the bioconversion of ICA into ICT, which is a cost-effective method with a high molecular conversion rate (91.2%) [21].

### 2.2. Medicinal Properties

Thanks to their outstanding medicinal properties in preventing and curing many common health issues, icariin and its derivates have garnered great interest in terms of drug development.

One of the most important medicinal properties of icariin and its derivates is its anti-cancer effects. Although a great decrease in mortality rate has been achieved with the most recent adjuvant chemotherapy regimens, the side effects and toxicity of this procedure are still notable. Hence, there is a high demand for the development of a mechanism-based nontoxic agent that could be applied during patients’ treatment [22]. ICS has shown great potential as a new anti-tumor agent, due to its ability to potentiate paclitaxel-induced apoptosis in melanoma cells. Compared to 5-Fu, a chemosynthetic anticancer drug, icariside II showed stronger anticancer activity on Hela cells, where its IC_50_ was 10 µM for ICS, while in the case of 5-Fu, IC_50_ = 31.1 µM [5]. The apoptotic mechanism was also proven on human prostate cancer cells, which demonstrated the great potential for ICS to be used as an effective cancer chemotherapeutic agent [23]. Animal experiments demonstrated that ICS successfully inhibited cervical cancer cell proliferation and promoted apoptosis; therefore, ICS is a promising agent for use in cervical cancer therapies [24]. The anti-leukemic activity of icariin was thoroughly studied and its potential in offering a new treatment option for patients was confirmed [25,26]. As the main metabolite of icariin, icariside II also showed anti-leukemic effects in vitro; consequently, new opportunities for further studies were created [27]. Recently, in vitro experiments proved that ICS is able to promote immune cells to kill the rapidly growing cancer cells [28]. In vivo studies showed a great reduction in the weight of mice liver carcinoma, which was achieved with ICS treatment. The reduction compared to 5-Fu was 50% greater [6]. Icariside II contributed to the anti-proliferative and anti-apoptosis effect of thalidomide. Therefore, in combination with clinically used drugs, it can have an enhanced therapeutic effect in the treatment of multiple myeloma [29].

Recent studies [30,31] highlighted the importance of icariin and its derivates in drug development and in auxiliary therapies to cure different cardiovascular diseases [32]. A 26-week course of therapy, administered to spontaneously hypertensive rats, proved that ICS successfully reduced blood pressure and improved the ventricular wall thickness [33].

Ever since the ancient Chinese era, *Epimedium* extract has been widely used as an aphrodisiac. In the past few years, many studies have investigated and proved the role of icariin and icariside II in the treatment of erectile dysfunction [34]. An improvement in erectile function was observed in spontaneously hypertensive rats, since icariin successfully decreased the content of endothelial microparticles in their blood, from 1.58% in the control group to 1.01% [35]. In vitro studies indicated that icariin could lead to increased testosterone secretion [36]. Further in vivo experiments proved that rats treated with an appropriate dose of icariin had a significantly (*p* < 0.001) higher testosterone level. This treatment also showed a slightly (*p* < 0.01) greater sperm count than in the control group. It was indicated that icariin contributes to improved testosterone and sperm production by gene regulation [37].

The degeneration or death of nerve cells can lead to neurodegenerative diseases (such as Alzheimer’s disease, Parkinson’s disease, Huntington’s disease, amyotrophic lateral sclerosis, and multiple sclerosis [38]), which so far are incurable. Icariside II showed an approximate efficacy to match that of donepezil, which is one of the few drugs approved by the Food and Drug Administration (FDA) to slow the worsening of the symptoms of Alzheimer’s disease [7]. Icariside II can be used in the treatment of excitotoxicity-related diseases, due to its potential as a protective agent for the hippocampal neurons [39]. Several studies have indicated that ICS showed promising results in the prevention [40] and treatment [41] of brain ischemic injury. The currently prescribed non-steroidal anti-inflammatory drugs are highly debated, due to their inconsistent results and toxic effects. Therefore, a new treatment was developed to increase the efficacy of the treatment and reduce its toxicity. ICS is also a possible protective non-toxic agent for acute neuroinflammation-related diseases [42].

Osteoporosis is a health condition that weakens bones by causing low bone mass and the deterioration of bone tissue [43]. Recent studies have shown that icariin and its derivates are potential drugs for postmenopausal osteoporosis treatment. In vivo experiments on ovariectomized rats showed that icariside II can promote the bone marrow stromal cells’ differentiation into osteoblasts and enhance osteoblast activity [44]. Recent studies showed that icariin treatment on fractured bones can accelerate healing [45,46]. In vivo experiments on different ages of rats with induced tibia fractures showed that icariin promoted fracture healing by activating the BMP-2/Smad5/Runx2 pathway [47].

It is necessary to highlight that, reportedly, ICA, ICS, and ICT showed low to no toxicity [4,20,48].

**Figure 1:**
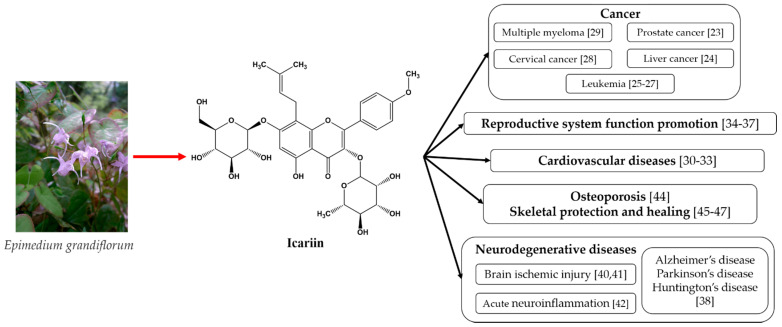
The medicinal properties of icariin [49].

The above-mentioned medicinal properties of icariin and its derivates are summarized in Figure 1.

As can be seen from Figure 1, icariin’s most well-known therapeutic properties are its anti-tumor effects, cardiovascular function improvement, sexual dysfunction amelioration, and neuroprotective effects. All of these properties emphasize the need for bioavailability improvements in these materials.

## 3. Bioavailability Improvements

For orally administered drugs, their key pharmacokinetic property is their bioavailability [50]. While icariin and icariside II showed good solubility in organic solvents such as ethanol, ethyl acetate, acetone, or chloroform [51,52], they are insoluble/minimally soluble in water [53]. Besides their poor aqueous solubility, their insufficient membrane permeability (~10^−7^ cm/s) contributes to their low oral bioavailability [54]. In the case of icariin, the oral bioavailability rate is only 12.02% [55].

**Figure 2:**
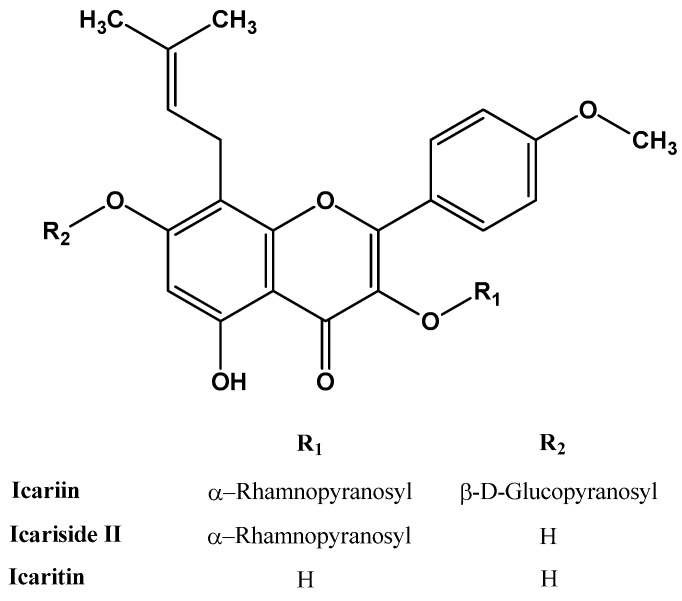
The molecular structure of icariin, icariside II, and icaritin.

As shown in Figure 2, the main difference between icariin and its derivates is at the R_1_ and R_2_ group levels. In the case of icariin, these groups are sugar moieties; due to their hydrophilicity, they are poorly absorbed from the human intestine [56]. Pharmacokinetic studies showed that hydrophobic particles are removed significantly faster from the bloodstream; conversely, the hydrophilic particles have a longer retention time. One of the possible reasons is that hydrophobic particles are promptly opsonized by plasma proteins [57].

One of the key parameters of bioavailability is sufficient drug aqueous solubility. The proportion of the drug that can enter the circulation is highly dependent on membrane permeability. Dissolved drugs are able to penetrate the aqueous layers of the biomembranes, thus entering the circulation [58]. Therefore, in the case of poorly water-soluble drugs, the absorption rate is minor and, as a consequence, the efficacy of the drug is also low.

In the past decade, numerous methods were developed to improve the poor oral bioavailability of flavonoids. Next, we present a comprehensive summary of the existing techniques of bioavailability enhancement of icariin and its derivates.

### 3.1. Pharmaceutical Technologies

Recently, carrier complexification gained more and more ground as an efficient way to enhance the therapeutic effect and bioavailability of bioflavonoids. This method is based on the complex formation of drug molecules with different carriers.

#### 3.1.1. Complex Formation with Phospholipids

Applying drug–phospholipid complexes (PC) is one of the viable strategies that can overcome the low oral bioavailability of drugs with poor aqueous solubility and biological membrane permeability [59]. In a study by Jin et al. [60], icariside II–phospholipid complexes were prepared by means of a reduction vaporization method. The different sizes of products resulting from this technique were tested in vivo on male rats. The results showed that the complexification successfully enhanced the bioavailability, and further improvements were achieved by reducing the particle size. Compared to the untreated ICS, an increment of 342% in the relative bioavailability, defined by the area under the curve (AUC_0–∞_), was observed. Additionally, PC-mixed micelles can be formed.

One of the possible materials that were recently applied in reference to ICA and its derivates is TPGS (d-α-tocopheryl polyethylene glycol 1000 succinate), also known as vitamin E TPGS [61]. Its lipophilic non-polar head and hydrophilic tail make it a feasible agent for micelle formation. In addition, TPGS has remarkable properties, such as configurable particle size and distribution, and presents high emulsifying capabilities [62]. The AUC_0–∞_ for the micelle ICS–phospholipid complexes was greater by 5.33 than the relative bioavailability of the plain ICS [63].

A different approach to achieving better bioavailability by means of PC is to improve its lipophilic properties, resulting in higher biomembrane permeability [64]. For example, in a study by Pan et al. [65], a viable method was used to prepare the ICA and ICS phospholipid complexes, that of wet media milling. Further improvements were achieved by a nanosuspension formulation. Although the aqueous solubility of the complexes remained unchanged, compared to the raw ICA and ICS, the oil solubility in *n*-octanol was 3.75 times greater. Consequently, the bioavailability of the complexes and complex nanosuspension was significantly increased.

#### 3.1.2. Complex Formation with Cyclodextrins

**Figure 3:**
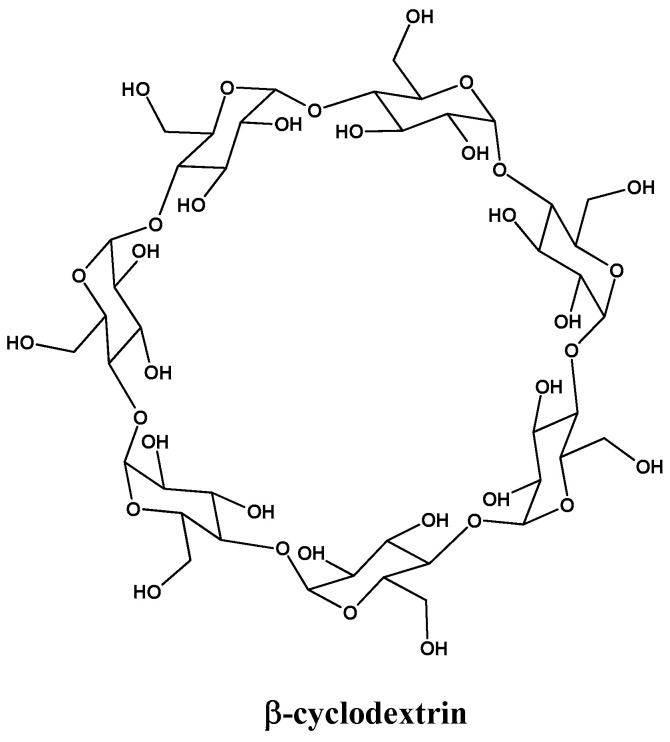
Molecular structure of β-cyclodextrin.

In the past few decades, complex formation techniques using cyclodextrins (CDs) have been profoundly investigated. CDs are oligosaccharides, as can be seen in Figure 3. They can form complexes with drugs, offering amazing properties such as improved water solubility, dissolution, stability, bioavailability, and taste-masking effects [66].

Reportedly, icariin complexes with β-cyclodextrin (ICA-β-CD) and hydroxypropyl-β-cyclodextrin (ICA-HP-β-CD) improved the intestinal absorption of ICA. The preparation of these complexes was achieved via a freeze-drying method. In vivo studies have proven that absorption can be highly affected by P-glycoprotein (Pgp). The ICA-HP-complex suppressed the activity of Pgp and increased its water solubility, consequently resulting in a great improvement in the intestinal absorption of ICA. In the case of the ICA-β-CD complex, it showed enhanced solubility but the treatment had no effect on Pgp [67].

The addition of CDs to ICA can result in a vast increase in its water solubility, consequently achieving considerably better bioavailability. It was proven that the aqueous solubility of ICA was successfully increased by almost 35 times at 60 °C and, correspondingly, by 28 times at 50 °C [68]. Another study showed that the complexation method with CDs resulted in solubility that was 36 times higher (525 g/mL) than that of the raw ICA [69].

To increase the bone regeneration effect of ICA, a new delivery system was investigated: the complexation of a β-cyclodextrin-alginate drug nanocarrier with icariin. This method resulted in higher efficiency in bone regeneration and repair [70]. Moreover, the uncontrolled burst release of ICA can be turned into a controlled burst release by the application of icariin-2-hydroxypropyl-β-cyclodextrin (ICA-2-HP-β-CD) inclusion complex-loaded PLLA scaffolds. These drug release studies compared the ICA with ICA-2-HP-β-CD inclusion complex-loaded PLLA scaffolds, which showed a significantly higher amount of drug release in the case of the complexes. The ICA had a total burst release of 21% in 5 days; meanwhile, in the case of the scaffold, the release was gradual, demonstrating 71% release in 5 days [71].

In a study by Mensah et al., the icariin/β–CD inclusion complex/bacterial cellulose (ICPBC) product was created in the form of hydrogels, which can be used in different fields, acting as target drug carriers, for wound dressings, or as facemasks in the cosmetics industry. In vitro studies showed the increased drug release (up to 47.9%) of icariin in the case of ICPBC [72].

CDs have some disadvantages. They can form aggregates, negatively affecting the water solubility [73]; in the case of intravenous administration, there is a risk of toxication because CDs cannot be hydrolyzed; and finally, they excrete into the kidneys. Despite this, their oral administration is safe because the CDs are metabolized in the colon [74].

### 3.2. Nanotechnologies

Nanotechnology can be used in drug development for the design, production, and application of structures, devices, and systems by controlling the size and material morphology in the nanometer range.

Nanotechnology can improve the pharmacokinetics of drugs in different ways [75]. Firstly, the absorption and delivery of drugs with low water solubility can be enhanced by means of encapsulation in nanocarriers [76]. This can simultaneously increase the chemical stability. Secondly, nanocarriers can protect the drugs from biodegradation or excretion, thereby improving the pharmacokinetic profile of the compounds [77]. Thirdly, the drug’s distribution and targeting can be enhanced [78]. However, nanomedicine compounds can be engineered to increase drug penetration and selectively redirect chemotherapy or targeting compounds to the tumor cells [79].

#### 3.2.1. Formation of Micelles

One of the strategies applied in the field of drug bioavailability improvement by means of nanotechnology is the application of a drug-loaded micelle mixture. These systems have garnered great interest due to their enhanced permeability, thus making them capable of high quantities of drug accumulation at the target site [80].

In a study by Rahdar et al. [81], icariside II-loaded binary mixed micelles were prepared with Solutol^®^ HS15 (polyoxyethylene-660–12-hydroxy stearate) and Pluronic^®^ F127 using the solvent evaporation method. These surfactants were selected because of their amazing properties, such as high bioavailability and low toxicity. F127 is capable of incorporating lipophilic drugs and creating a hydrophilic shell. Consequently, it can form micelles in aqueous solutions. This means that it can function as a membrane-stabilizing agent and cell encapsulation material. HS15 can be divided into two parts, the lipophilic part, consisting of polyglycol monoesters and diesters of 12-hydroxystearic acid, and the hydrophilic part, which is a free polyethylene glycol [82]. ICS-mixed micelles with HS15:F127 in a 4:1 mole ratio showed the highest bioavailability. Subsequent pharmacokinetic studies showed that the AUC_0–∞_ of those micelles was 317% greater than that of the ICS. This higher relative bioavailability is achieved due to the increased efflux ratio. Moreover, the micelles form in a smaller diameter and show larger dispersion, thus improving the solubility, while HS15 increases the permeability of the intestinal epithelium [83].

In a study by Yan et al. [84], the ICS-loaded micelles were formed with didecyldimethyl ammonium bromide (DDAB) and d-α-tocopheryl polyethylene glycol succinate (TPGS). Additionally, hyaluronic acid (HA) was also added to the mixture. Thus, the two obtained mixtures are the DTBM, which consists of ICS–DDAB–TPGS, and the HDTBM, which consists of ICS–DDAB–TPGS–HA, both being mixed micelles. The DDAB is a surface-active agent, a surfactant that is used as a surface stabilizer due to its capability of reducing the interfacial tension between the aqueous environment and hydrophobic surfaces [85]. In addition, it has been reported that DDAB can induce the death of a variety of tumor cells [86]. HA is used as a drug carrier due to its useful properties, such as targeted delivery and maintaining a high dose over a long period [87]. The ICS-loaded micelles were prepared by means of the thin-film hydration method. The pharmacokinetic studies showed that while ICS had a fast release (4 h), the mixed micelles had a controlled slower release (over 24 h). The in vivo studies revealed that HDTBM had a significantly higher antitumor efficacy than the free ICS [84].

The micelle formation technique has been proven to be an effective way to increase the bioavailability of drugs. In numerous cases, it was reported [61,63] as being used in combination with other methods to achieve a better increment of water solubility and higher drug uptake.

In a study undertaken by Han et al. [88], a self-assembled micelle between poly (ethylene glycol)-poly (L-lactic acid) (PEG-PLLA) and poly (D-lactic acid)-poly(N-isopropylacrylamide) (PDLA-PNIPAM) was used as a carrier for icariin. The relative bioavailability of the polymeric micelles, pure ICA, and Pluronic F127-ICA micelles was measured. The results indicated an increment of 500% compared to that of ICA and of 216% compared to the F127-ICA micelles.

#### 3.2.2. Nanocarriers

Nanocarriers are widely used to transport drugs to their target site. In the case of icariin and its derivates, in a study by Ming et al. [52], exosomes showed amazing properties in promoting osteoblast proliferation. In this study, fetal bovine serum (FBS) was the nanocarrier used to transport the icariin. The FBS successfully incorporated the ICA and significantly improved the proliferation of cells, compared to the plain ICA.

#### 3.2.3. Nanogels

Some of the most recently studied nanocarriers are nanogels. They have attracted attention recently due to their capability of efficiently delivering the drugs to the target site with small to negligible adverse effects [89]. By optimizing their molecular composition, size, and morphology, nanogels can be tailored to react to environmental changes, thus ensuring space- and stimulus-triggered drug release in vivo [90].

A reportedly self-assembled thermosensitive hydrogel system (icariin-NGSTH) was developed to increase the intranasal delivery of icariin. These hydrogels were prepared by a reverse microemulsion method using Span 80 and Tween 80. In vivo studies with mice and a chronic unpredictable mild stress model showed that the icariin-NGSTH had a remarkably swift antidepressant effect [91].

In a study by He et al. [92], hyaluronic acid–icariin hydrogels (HA-ICA) were studied. The fabrication of this hydrogel started with the preparation of methacrylic anhydride–icariin (MA-ICA) and methacrylic anhydride–hyaluronic acid (HAMA). The HA-ICA hydrogel was obtained by means of photopolymerization of the HAMA and MA-ICA suspension. In the case of the HA-ICA hydrogels, the burst release of ICA was mitigated; therefore, the long-lasting bioactivity of the hydrogels was successfully achieved.

#### 3.2.4. Nanocrystals

Reportedly, icaritin nanocrystals were achieved in a study by Yan et al. using the antisolvent-precipitation method [54]. The icaritin nanosuspensions had a uniform size distribution of ~200 nm, which led to an increment of the specific surface area; consequently, oral bioavailability was successfully enhanced. In vivo studies proved that the relative bioavailability of the product was doubled compared to raw icaritin [54].

#### 3.2.5. Microspheres

In a study undertaken by Hua et al. [93], gelatin/hyaluronic acid–icariin microspheres were fabricated and glutaraldehyde was used as a cross-linker. The purpose was to regulate the release of icariin by controlling the contents of the icariin and cross-linker. It was demonstrated that the hydrogels containing the icariin had a rougher surface; by modifying the content of the cross-linker, the encapsulation efficiency could be regulated. The release speed of icariin is proportional to its quantity in the matrix, i.e., lower levels of icariin result in a slower release. This method can be used to achieve a slower release of the drug [93].

#### 3.2.6. Extracellular Vesicles

Extracellular vesicles (EVs) are membranous nanoparticles that can be obtained from various living cells [94]. Recently, great interest has been expressed in EVs due to their wide variety of therapeutic applications, since they can be used as effective drug carriers. One of the greatest advantages of EVs, when they are used as natural carriers, is that they can load both hydrophobic and hydrophilic drugs [95].

In a study by Tan et al. [96], icariside II was loaded in bovine milk extracellular vesicles (BM-EV) using incubation and sonification methods. The pure ICS was compared with products created using different methods. The BM-EV loaded with ICS showed improved efficacy. As a consequence of its low encapsulation efficiency (~1%), this method requires further improvements, such as the optimization of the encapsulation.

#### 3.2.7. Solid Lipid Nanoparticles

In the last few decades, great interest has been given to solid lipid nanoparticles as drug delivery systems because of their ability to efficiently deliver hydrophobic bioactive compounds [97]. Those particles can significantly reduce the mobility of the bioactive compounds in the lipid matrix, preventing coalescence. This results in higher stability and decreased drug swelling into the emulsifier, consequently ensuring continuous drug release [98].

In a study carried out by Liu et al., lyophilized icariin stealth solid lipid nanoparticles (ICA-SSLN) were fabricated to improve the bioavailability of ICA. The ICA-SSLN was compared to an ICA control solution (ICA-Sol). The pharmacokinetic studies emphasized that the solid lipid nanoparticles successfully lengthened the half-life of ICA in blood and increased its relative bioavailability. Moreover, the drug uptake in the kidney was greatly increased [99].

### 3.3. Structural Transformation

Today, the polymorphism and isostructurality of solid materials play an important role in the fields of medicine and food supplements. Different polymorphs of a solid bioactive agent may demonstrate different physicochemical properties, such as enthalpy of fusion, dissolution behavior, and stability. Thus, their bioavailability can differ [100]. Meanwhile, polymorphism means that chemical substances can have different crystalline modifications, and the isostructurality forms an identical packing arrangement [101].

In a study by Lina et al. [102], two polymorphs in pure form and ten different solvate modifications of icariin were obtained; among these was also an α anhydrous form. Compared to ICA, this form showed improved characteristics, such as better thermal stability, lower hygroscopicity, and higher solubility in water.

### 3.4. Absorption Enhancement

Absorption enhancers are a widely used method for increasing the water solubility of poorly soluble drugs. The better solubilization of those bioactive compounds leads to increased intestinal absorption, thus improving their bioavailability [103]. The flavonoids can be hydrolyzed into their secondary glycoside and aglycone forms. In this case, the icariin in the first step can be hydrolyzed into icariside II and, subsequently, into its aglycone form, icaritin (ICT), which has enhanced bioavailability compared to icariin [54].

According to the study undertaken by Congyan et al. [104], the absorption of icariin can be enhanced with the addition of snailase. Reportedly, this is composed of over 20 different kinds of enzymes (cellulase, pectinase, etc.) [105]. In vivo study using rats with osteoporosis showed that the hydrolysis rates of the icariin and its derivates were highly reduced [106], which led to an efficacy decrement of these bioactive compounds. One of the techniques used to solve this problem is to increase intestinal hydrolysis with the addition of snailase. In the research published by Congyan et al. [104], the icariin and snailase were packed into enteric-coated capsules, and the authors studied their pharmacokinetics in vivo on rats. The results highlighted that this method successfully increased the bioavailability of icariin by 50% in the case of rats with osteoporosis; however, no significant improvement was seen in the case of normal rats.

#### Colon-Specific Drug Delivery

Colon-directed drug delivery systems are a widely investigated area in terms of designing selectively releasing drugs in the colonic environment, enhancing the oral delivery of active drugs to cure colonic diseases [107]. These systems can provide on-site treatment at the disease location, enabling a reduction in the dosage of the drugs, thus diminishing their potential side effects. In many cases, the upper digestive tract can cause enzymatic degradation or other chemical damage to the bioactive agent (proteins, peptides, etc.), thus reducing their bioavailability [108].

In a study by Qiang et al., alginate-chitosan microspheres loaded with icariin were used as a colon-specific delivery system. Polysaccharides such as chitosan and alginate can withstand the low pH in the stomach and efficiently release the drug in the colon, where the environment has a higher pH. In vitro release studies showed that 10% of the icariin was released in simulated gastric fluid, while 65% was released in simulated colonic fluid [109]. Therefore, this technique successfully delayed the release of icariin in the upper gastrointestinal tract until it reached the colon.

A summary of the above-mentioned methods and their efficiency is given in Table 1. As can be seen, the most substantial improvements were achieved by the complex formation and micelle formation techniques. However, some of these methods are still under development and with further improvements, great results may be achieved. Other methods to achieve an efficiency improvement in this field, besides increasing the bioavailability, are related to the controlled release of the drug.

**Figure 4:**
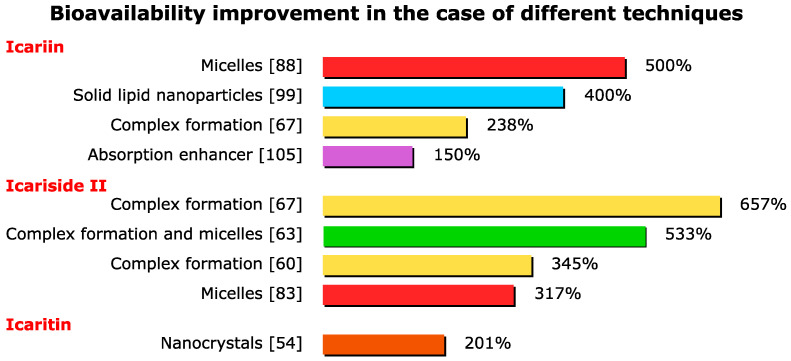
Bioavailability improvement in the case of different techniques.

As can be seen in Figure 4, by far the highest bioavailability improvements were achieved by means of the complex formation and micelle formation technique. The synergistic effect between the micelles and complex formation also resulted in great improvements in efficiency.

## 4. Conclusions and Future Prospects

This paper surveyed an extensive array of literature that discussed bioavailability improvement strategies for icariin and its derivates. The currently available evidence in this review proved that these flavonoids have great potential in treating many common health issues. Several methods were studied to increase the bioavailability of these therapeutic agents; in the most successful, a 6.57-fold bioavailability improvement was achieved using phospholipid complex formation. It is noteworthy to mention that the micelle formation methods also resulted in great improvements in bioavailability. Applying several different techniques at the same time may result in a synergistic effect, thus exponentially improving the bioavailability of poorly soluble flavonoids.

There is great potential for further improvements using the currently available evidence on this topic, alongside an urgent need for more studies to be conducted in the future.

## Figures and Tables

**Table 1:** Summary of the bioavailability improvement methods and their efficiency in the context of icariin, icariside II, and icaritin.

Flavonol	Technique	Preparation Method	Carrier	Efficiency Improvement	Reference
Icariin	Complex formation	Lyophilization	β-Cyclodextrin	Cumulative drug release: 62%	[72]
	Complex formation	Lyophilization	β-Cyclodextrin	Solubility (water, 25 °C): 1.68-fold Absorption rate: 2.32-fold Permeability rate: 3.46-fold	[67]
	Complex formation	Saturated solution method	β-Cyclodextrin	Solubility (water, 37 °C): 36-fold	[69]
	Microspheres	Emulsion and coagulation	Gelatin and hyaluronic acid	Controlled release	[93]
	Nanocarriers	Mixing and centrifuging	Fetal bovine serum exosomes	Cell proliferation significantly increased (*p* < 0.001)	[52]
	Micelle	Mixing and vacuum-drying	PEG-PLLA/PDLA-PNIPAM polymers	Bioavailability: 5-fold	[88]
	Solid modification	Heating	-	Solubility (water, 25 °C): 1.5-fold	[102]
	Hydrogel formation	Photopolymerization	Hyaluronic acid	Controlled release	[92]
	Hydrogel formation	Reverse microemulsion method	Span 80 and Tween 80	-	[91]
	Solid lipid nanoparticles	High temperature melt-cool solidification method	Liposomal vesicles	Bioavailability: 4-fold	[99]
	Absorption enhancer	Mixing	Snailase	Bioavailability: 1.5-fold	[104]
Icariside II and icariin mixture	Complex formation	Wet media milling	Soybean phospholipids	ICA: Dissolution: 1.39-fold (2 h) Bioavailability: 2.38-fold ICS: Dissolution: 1.24-fold (2 h) Bioavailability: 6.57-fold	[65]
Icariside II	Complex formation	Reduction vaporization	Phospholipid	Bioavailability: 3.45-fold	[60]
	Complex formation and micelles	Solvent evaporation	Phospholipid and vitamin E TPGS 1000	Bioavailability: 5.33-fold	[63]
	Micelles	Thin film hydration	DDAB and TPGS with hyaluronic acid	Controlled release	[84]
	Micelles	Solvent evaporation	Solutol^®^ HS15 and Pluronic F127	Solubility (water, temperature not mentioned) 900-fold Bioavailability: 3.17-fold	[83]
	Nanocarriers	Incubation and sonification	Bovine milk as extracellular vesicles	-	[96]
Icaritin	Nanocrystal	Antisolvent-precipitation	Hydroxypropyl methylcellulose as stabilizer	Bioavailability: 2.01-fold	[54]

## Data Availability

No new data were created or analyzed in this study. Data sharing is not applicable to this article.
